# Deep learning-based approach for Arabic open domain question answering

**DOI:** 10.7717/peerj-cs.952

**Published:** 2022-05-04

**Authors:** Kholoud Alsubhi, Amani Jamal, Areej Alhothali

**Affiliations:** Department of Computer Science, Faculty of Computing and Information Technology, King Abdulaziz University, Jeddah, Saudi Arabia

**Keywords:** Arabic open domain question answering, Transformer-based models for question answering, Dense information retrieval approach

## Abstract

Open-domain question answering (OpenQA) is one of the most challenging yet widely investigated problems in natural language processing. It aims at building a system that can answer any given question from large-scale unstructured text or structured knowledge-base. To solve this problem, researchers traditionally use information retrieval methods to retrieve the most relevant documents and then use answer extractions techniques to extract the answer or passage from the candidate documents. In recent years, deep learning techniques have shown great success in OpenQA by using dense representation for document retrieval and reading comprehension for answer extraction. However, despite the advancement in the English language OpenQA, other languages such as Arabic have received less attention and are often addressed using traditional methods. In this paper, we use deep learning methods for Arabic OpenQA. The model consists of document retrieval to retrieve passages relevant to a question from large-scale free text resources such as Wikipedia and an answer reader to extract the precise answer to the given question. The model implements dense passage retriever for the passage retrieval task and the AraELECTRA for the reading comprehension task. The result was compared to traditional Arabic OpenQA approaches and deep learning methods in the English OpenQA. The results show that the dense passage retriever outperforms the traditional Term Frequency-Inverse Document Frequency (TF-IDF) information retriever in terms of the top-20 passage retrieval accuracy and improves our end-to-end question answering system in two Arabic question-answering benchmark datasets.

## Introduction

Throughout the history of Natural Language Processing (NLP), OpenQA has remained a long-standing issue. OpenQA is an intelligent system that answers questions based on large-scale data. The data can be in a structured form (*e.g*., knowledge bases), semi-structured form (*e.g*., tables), and unstructured form (*e.g*., open textual content). Since 1999, when the National Institute of Standards and Technology (NIST) first included the QA track in the Text Retrieval Conference (TREC) contests, OpenQA research has exploded (Chen et al., 2017).

Question Answering (QA) research has received considerable attention in recent years due to the importance of QA applications. Traditional QA methods are often performed in three stages first, analyze the question, retrieve the relevant articles, and then extract the answer (Zhu et al., 2021). Reading comprehension tasks have progressed to offer QA researchers a simple two-stage framework. In the first stage, a passage retriever returns a subset of passages that include some of the answers to the question. While in the second stage, a passage reader analyzes the retrieved passages to extract the correct answer (Chen et al., 2017).

The passage retrieval task often uses classical Information Retrieval (IR) approaches such as TF-IDF (Sammut & Webb, 2017) and BM25 (Amati, 2009) to retrieve relevant candidates passages. Recent passage retrieval approaches have used deep learning techniques to replace classical IR approaches. They, in particular, utilize dense representations which learn to encode questions and documents into a latent vector space where text semantics beyond term match can be measured (Zhu et al., 2021). The dense passage retrieval models work by feeding dense representations of a question and passages into a language model. The most relevant passages are then ranked using the dot-product of these two representations. The passage reading task in the OpenQA system aims to infer the answer to a question from a set of candidate passages. This task is more complicated than the original Machine Reading Comprehension (MRC) that takes only a passage and corresponding question to extract the answer. To understand the progress in the passage reading task, one needs to have some background about transformer-based pre-trained NLP models such as the Bidirectional Encoder Representations from Transformers (BERT) (Devlin et al., 2019) that have contributed heavily to the success of many NLP applications (Hedderich et al., 2021). Similar to other NLP tasks, pre-trained transformer-based models, more specifically BERT-based models, have been successfully utilized in many English QA systems (Qiu et al., 2020). However, few studies have investigated the effects of using pre-trained models for Arabic QA tasks, despite the availability of several Arabic pre-trained transformer models, such as AraBERT (Antoun, Baly & Hajj, 2020) and AraELECTRA (Antoun, Baly & Hajj, 2021).

In this paper, we contribute to improving the performance of the Arabic OpenQA system. We implement a two-stage (Retriever-Reader) architecture which is the most efficient and promising way to create OpenQA systems (Huang et al., 2020). We use deep learning techniques to build the information retriever and reading comprehension models. To create our OpenQA system, we first fine-tuned the Dense Passage Retrieval (DPR) (Karpukhin et al., 2020) using the ARCD (Mozannar et al., 2019) and TyDiQA-GoldP (Clark et al., 2020) datasets. Then, we connected the DPR with the AraELECTRA passage reader. Finally, we compared the performances of the OpenQA on different benchmark datasets.

In this paper, “Related works” presents studies related to OpenQA and transformer-based QA; “Model Overview” presents details of the model; “Dataset” provides details of the datasets used in our experiments; “End-to-End System: Arabic OpenQA” presents our end-to-end OpenQA system; and finally “Experiments and Results” covers the experiments and evaluations of the system.

## Related works

Modern OpenQA systems combine IR and neural MRC models to answer open-domain factual questions. The IR system’s goal is to find and rank relevant passages likely to contain the correct answers to natural language questions. Traditional IR approaches incorporate the sparse representation approach (TF-IDF or BM25) to rank articles based on the weighted similarity score between documents and questions. However, in recent years, several dense representation-based IR approaches have been developed that learn to encode questions and passages into a latent vector space where text semantics beyond term match can be measured.

Dense approaches outperform traditional sparse retrieval methods due to their ability to capture lexical or semantic similarities, not only matching keywords (Karpukhin et al., 2020). Karpukhin et al. (2020) focused on creating the correct dense embedding model using only pairs of questions and answers by combining the BERT pre-trained model and a dual-encoder architecture. Their dense passage retriever uses a dense encoder to convert any text into a dimensional real-valued vector and creates an index for all passages to be retrieved. Their proposed model achieved better results than multiple open-domain QA on many QA datasets, including SQuAD, Natural Questions, and TriviaQA.

Lee, Chang & Toutanova (2019) designed a QA model in a supervised manner, where the retriever and reader are trained together to optimize the marginal log-likelihood of the right answers. There is no specific IR system for this QA model. Instead, the model can retrieve any text from a *corpus* that is open to the public. The ORQA system only requires (question, answer) string pairs during the training, rather than ground-truth context passages (*i.e*., reading comprehension datasets). The retriever and reader components were designed using BERT.

Guu et al. (2020) proposed an effective approach that combines a learned textual neural knowledge retriever with the language model pre-training methods. Unlike models that store knowledge in their parameters, this approach directly highlights the role of world knowledge by requiring the model to choose which knowledge to extract and employ during inference. The language model uses the retriever to retrieve documents from Wikipedia before making each prediction, and then the documents are examined. On three QA benchmarks, the Guu et al. (2020) model outperformed all the previous models, even when compared to state-of-the-art models.

Neural MRC models offer a powerful solution for answer extraction in OpenQA, eliminating the need for traditional linguistic analytic techniques and revolutionizing OpenQA systems. Neural MRC models utilize the pre-trained language models for QA tasks in a self-supervised manner. In Yang et al. (2019), BERT was integrated with the open-source Anserini information retrieval toolkit to create an end-to-end question answering system. Unlike multi-stage retrieval systems, which retrieve documents first and then rank the retrieved passages, they employed a single-stage retriever to identify Wikipedia text segments to send directly to the BERT reader. They fine-tuned BERT to remove the final SoftMax layer over several answer spans.

Several researchers have used ELECTRA (Efficiently Learning an Encoder that Classifies Token Replacements Accurately) for OpenQA reading comprehension. ELECTRA is a self-supervised language representation learning approach pre-trained on a large *corpus*. Antoun, Baly & Hajj (2021) developed the pre-training text discriminators for the Arabic language understanding named AraELECTRA. The discriminator network has the same architecture and layers as a BERT model. To fine-tune their approach, they added a linear classification layer on top of ELECTRA’s output and fine-tuned the whole model with the added layer on reading comprehension tasks. Researchers evaluated the model on many Arabic NLP tasks, including reading comprehension. Compared to QA in the English language, the progress in Arabic language QA systems is very slow. This is due to the shortage of NLP resources and datasets for Arabic QA. Arabic OpenQA research incorporates the sparse approach for passage retrieval. SOQAL (Mozannar et al., 2019) was the first attempt at developing modern Arabic OpenQA systems, and it was created by integrating hierarchical TF-IDF traditional IR approaches with a Multilingual Pre-trained Bi-directional Transformer (mBERT) neural MRC model to answer open-domain factual queries. The system gets a set of documents relevant to the query, retrieves the text most linked to the user’s query, and returns the text as an answer using mBERT. An Arabic Reading Comprehension Dataset (ARCD) with 1,395 questions in diverse fields was created based on Wikipedia articles. The ARCD dataset experiment with the BERT-based reader achieved a 50.10 F1-score, and the experiment on the Arabic-SQuAD dataset achieved a 48.6 F1 score. The overall performance on the top five answers was 20.7 EM and a 42.5 F1 score.

Ahmed, Bibin & Babu Anto (2017) proposed a model called question answering based on neural networks to answer factoid questions by accessing a knowledge base. The system consisted of a question analyser, a knowledge retriever, and an answer generator. The question is represented as a vector in the question analyser module using a bidirectional Gated Recurrent Unit (GRU). The retrieved facts and the short-term memory of the recurrent neural network are used to generate the answer. The accuracy of their system was tested using a knowledge base, and the results showed a 53% accuracy rate.

Ahmed, Ahmed & Babu Anto (2017) proposed an Arabic QA based on machine-learning techniques for question classification and answer selection tasks. Their system consisted of a question-analysis module that included a tokenizer, a stemmer, stop-word removal, and a question class identifier. Then, the passage retrieval module returned related passages from the document set. The last component was the answer extraction. They used a Support Vector Machine (SVM) classifier for question classification and answer selection. They tested their system using a set of 434 translated questions from TREC-QA Track, and the MRR score was 57.7%.

Ahmed & Babu Anto (2016) proposed an Arabic QA system that answers two types of questions: “how” and “why” questions. The system consisted of question analysis, question expansion, document retrieval, and answer extraction. They used TF-IDF weighing to retrieve the related documents from the *corpus*. The F1 measure was 56% for the “how” questions and 64% for the “why” questions.

Almiman, Osman & Torki (2020) discussed the Arabic community question answering problem. They used different types of similarity features and studied the effect of using preprocessing. They produced a novel deep neural network ensemble model from the semantic and lexical similarity features. The model utilized recent advances in language models using the BERT model. The model achieved an MRR value of 68.86%.

## Model overview

The OpenQA problem is formulated in this study with the retriever-reader approach that consists of two major modules. The first is the dense passage retriever which, given a large *corpus* (*e.g*., Arabic Wikipedia), retrieves a passage or several passages that likely contain the correct answer to a given query. The second is the passage reader, which is a neural MRC model that finds the answer from the retrieved passages. The following subsections provide more details about both modules.

### Dense passage retriever

Dense Passage Retrieval (DPR) was introduced in 2020 by Karpukhin et al. (2020) for open-domain QA tasks as an alternative to the TF-IDF, and BM25 passage retrieval approaches. This retriever improves the reader by working as a lightweight filter reducing the number of documents that must be processed. Dense methods, such as a dual-encoder in DPR, have outperformed the sparse techniques in English open-domain QA. These methods use deep neural networks to embed both the document and the question into a shared embedding space. The dense model uses transformer-based encoders that are more sensitive to characteristics such as lexical variations or semantic relationships, whereas the sparse methods consider the text as a “bag of words,” without considering word order and grammar (Karpukhin et al., 2020).

DPR uses two independent BERT encoders to train a retriever using pairwise questions and answers. To train DPR, we need a question, their answer, positive passages, and negative passages. The negative samples can be obtained using methods such as BM25 to return negative samples to a question from the *corpus* that does not contain the correct answer or the in-batch negatives method to return samples that are paired with other questions in the same batch. The representation-based method used in DPR can be very fast since passages can be calculated and indexed offline in advance. However, because the representations of the question and passage are generated independently, only shallow interactions between them are captured, which may reduce retrieval efficiency. The experiments on this method show that the inner product function is the best way to calculate a dual-encoder retriever’s similarity score.

This allows the DPR model to capture the lexical or semantic similarities. Thus, phrases that contain different tokens (keywords), but the same meaning may still be mapped to vectors that are located relatively close to each other. For example, the DPR would be able to better match (“نقود” with “مال” - money) and extract the correct context. The purpose of DPR is to index all passages in a low-dimensional, continuous space so that it may efficiently retrieve the top passages relevant to the input question for the reader at run-time (Karpukhin et al., 2020).

#### Dense passage retriever methodology

**Figure 1:**
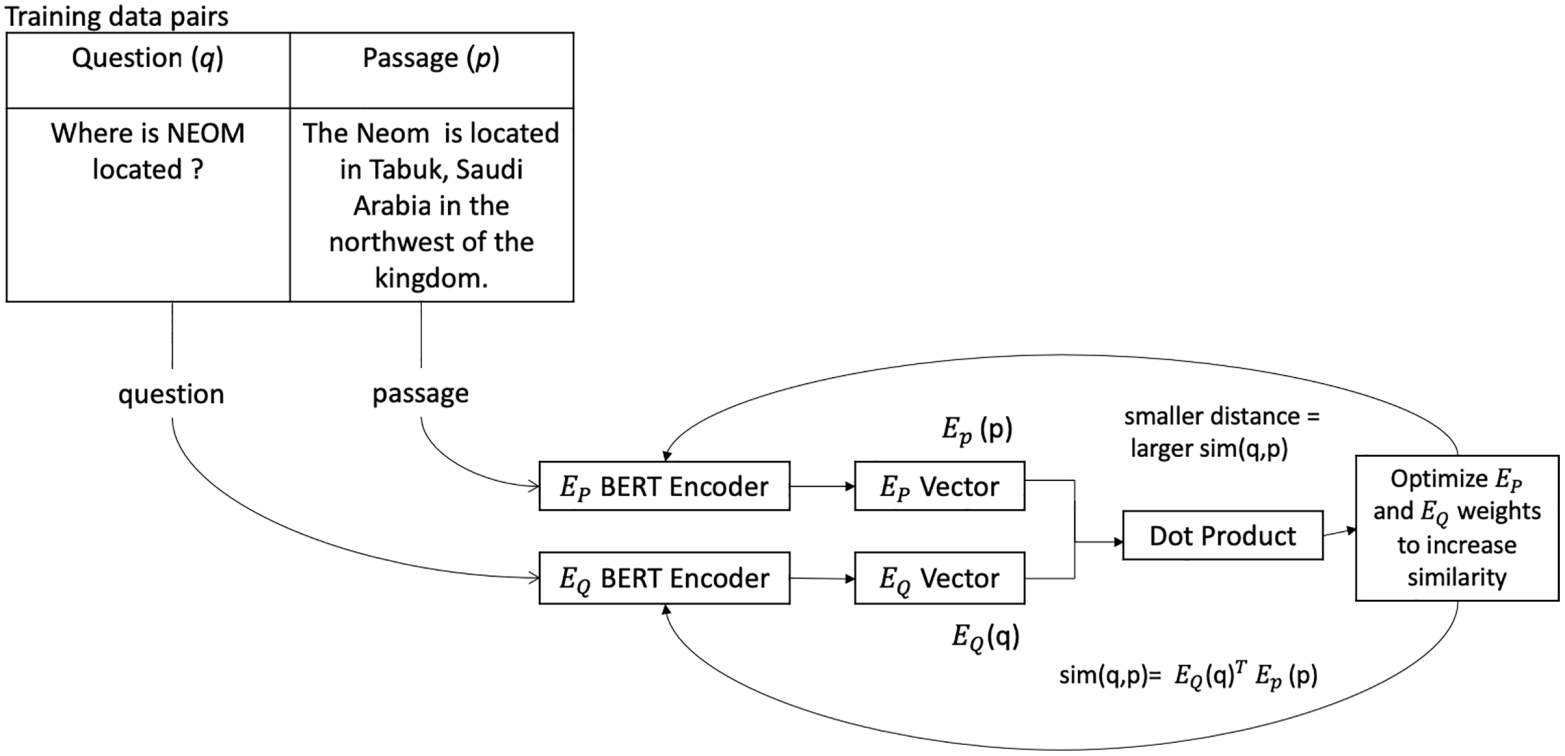
The process of data flow through a DPR model during training. Where *E_P_* is the passage encoder, and *E_Q_* is the question encoder adapted from Briggs (2021).

DPR is an efficient retrieval method that uses dense representations to compute relevancy. Dense techniques use text as the input to neural network encoders, and the text is represented as a vector of a fixed size, usually 768. Despite the fact that the individual dimensions do not match any specific language or linguistic feature, each dimension stores some information about the text. The relative density of these vectors is due to the rarity of a zero value in them. The model architecture used a dual encoder, that is, two BERT base models, one to encode the query and the other to encode the passage. The dot product similarity between the query and the document embeddings is used to rank documents. The passage encoder is used in all the passages and indexes them using FAISS (Johnson, Douze & Jegou, 2021). During training, the question-context pair is sent into the DPR model, and the weights are tuned to maximize the dot product between the two model outputs. The dot product value of the two model outputs measures the similarity between both vectors. A higher dot product correlates to higher similarity. The context encoder and the question encoder were both trained to give very similar vectors as output for relevant question-context pairs (see Fig. 1). Separate encoders will help with queries shorter than documents, and employing "in-batch negatives," gold labels used as negative examples for other data in the same batch, is more effective.

DPR (Karpukhin et al., 2020) employs a dense encoder *E*_*P*_(·), which converts any text into a *d*-dimensional vector and generates an index for all *M* passages. DPR uses a separate encoder called *E*_*Q*_(·) during run-time, which converts the input question to a d-dimensional vector and returns *K* nearest passages vectors to the question vector. Using the dot product of their vectors, the similarity between the question *q* and the passage *p* can be defined as the following (Karpukhin et al., 2020):

(1) $$sim(q,p) = {E_Q}{(q)^{\tt T}}{E_P}(p)$$

The DPR encoder outputs the representation at the unique initial token of the sequence [CLS] by combining two independent BERT networks (base, uncased). When training the DPR, the training data consists of *m* examples. Each example includes one question (*q*_*i*_) and one related positive passage $(p_i^ + )$ along with *n* unrelated negative passages $(p^{-}_{i,1})$. The training data can be formulated as follows: ${\cal D} = \big\{ {\big\langle {{q_i},p_i^ + ,p_{i,1}^ - , \cdots ,p_{i,n}^ - } \big\rangle } \big\}_{i = 1}^m$ (Karpukhin et al., 2020) and the optimizing loss function for the negative log-likelihood of the positive passage as follows (Karpukhin et al., 2020):

(2) $$\matrix{{L\left( {{q_i},p_i^ + ,p_{i,1}^ - , \cdots ,p_{i,n}^ - } \right) = } & { - \log \displaystyle{{{e^{{\rm sim}\left( {{q_i},p_i^ + } \right)}}} \over {{e^{{\rm sim}\left( {{q_i},p_i^ + } \right)}} + \sum_{j = 1}^n {e^{{\rm sim}\left( {{q_i},p_{i,j}^ - } \right)}}}}} \cr }$$

#### Retriever evaluation

The main metrics used in IR evaluations are the recall, the precision, the accuracy, and the Mean Average Precision (MAP) (Teufel, 2007). The fraction of relevant documents that are retrieved is described as the recall (see Eq. (3)). Precision is the fraction of retrieved documents that are relevant (see Eq. (4)). The proportion of correctly classified documents, whether relevant or irrelevant, is defined as the accuracy (see Eq. (5)). The Mean Average Precision (MAP) for a set of queries is the average of each query’s average precision scores(see Eq. (6)). MAP is a value that ranges from zero (no matches) to one (the system found correct documents for all top results). It’s extremely helpful when there’s more than one correct document to find.

(3) $${\rm Recall = }\displaystyle{{\text{Number of relevant documents retrieved}} \over {\text{Number of relevant documents}}}$$

(4) $${\rm Precision = }\displaystyle{{\text{Number of relevant documents retrieved}} \over {\text{Number of retrieved documents}}}$$

(5) $${\rm Accuracy = }\displaystyle{\text{Number of relevant retrieved and irrelevant documents not retrieved} \over {\text{Total number of all documents}}}$$

(6) $$\text{Mean Average Precision (MAP)} = \displaystyle{{\sum_{q = 1}^Q {\rm AveP }({\rm q})} \over Q} \text{where Q is the number of queries}.$$

### AraELECTRA passage reader

ELECTRA (Efficiently Learning an Encoder that Classifies Token Replacements Accurately) is a new and more efficient self-supervised language representation learning approach. ELECTRA, similar to the Generative Adversarial Network (GAN) (Zhang et al., 2018), trains two transformer models, the generator and discriminator. The model performs a pre-training task called Replaced Token Detection (RTD) to replace some tokens with plausible alternatives sampled from a small generator model. In doing this, the discriminator model tries to predict whether a token is an original or a replacement by a generator sample instead of training a model to predict the identities of the masked tokens. ELECTRA was initially released as three pre-trained models: small, base, and large. ELECTRA achieved state-of-the-art results on the SQuAD2.0 dataset in 2019 (Clark et al., 2020).

#### AraELECTRA

**Figure 2:**
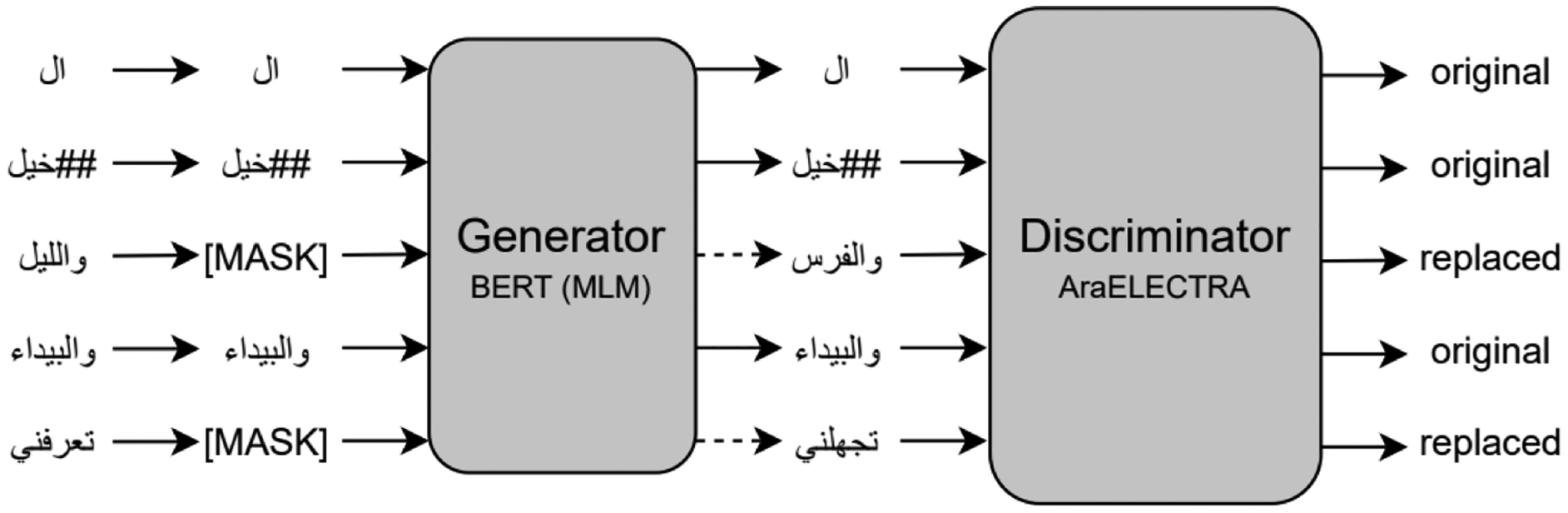
Replaced token detection pre-training approach (Antoun, Baly & Hajj, 2021).

AraELECTRA is an Arabic language representation model pre-trained using the RTD (Antoun, Baly & Hajj, 2021) methodology on a large Arabic text *corpus*. AraELECTRA consists of 12 encoder layers, 12 attention heads, 768 hidden sizes, and 512 maximum input sequence lengths for a total of 136 million parameters. Figure 2 shows the replaced token detection pre-training task for AraELECTRA.

#### Reader evaluation

We evaluated our model based on two metrics that are commonly used in QA tasks. The first is the exact match (EM), and the second is the F1-score.

**F1 METRICS: **The F1 score is a widely used metric in QA tasks. It is useful when both precision and recall need to be considered when evaluating the model performance. It is calculated by the individual words in the prediction against those in the correct answer. Precision is the ratio of correctly predicted tokens divided by the number of all predicted tokens. The recall is also the ratio of correctly predicted tokens divided by the number of ground truth tokens. If a question has many answers, then the answer that provides the highest F1 score is considered to be the ground truth.

(7) $$F1 = 2*\displaystyle{{{\rm Precision * Recall}} \over {{\rm Precision + Recall}}}$$

**Exact Match: **This is a true/false metric that measures each question-answer pair. If the predictions match the correct answers exactly, then the EM = 1 or else the EM = 0.

(8) $$\matrix{ {EM = \displaystyle{{\sum_{i = 1}^N F\left( {{x_i}} \right)} \over N},{\rm where}\;F\left( {{x_i}} \right) = \left\{ {\matrix{1,  {\rm if}\;{\rm predicted}\;{\rm answer} = {\rm correct}\;{\rm answer} \cr 0,  {\rm otherwise} \cr } } \right.} \cr }$$

## Dataset

**Figure 3:**
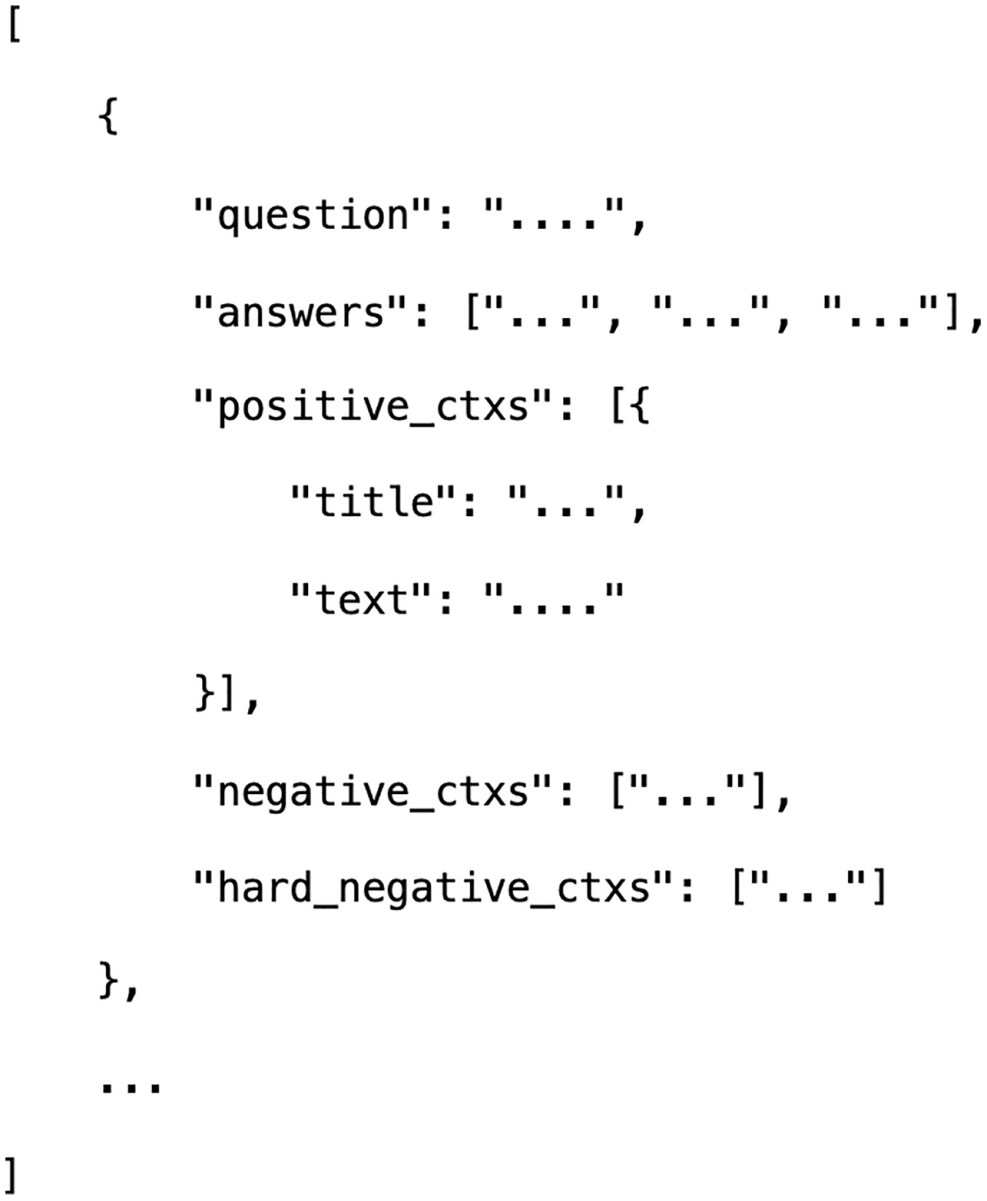
The dataset Structure for DPR. Where positive_ctxs: passages that are relevant to the question, negative_ctxs: this was used by the original DPR authors to compare it against the in-batch negatives approach and not used in our DPR so, we set it to an empty list, hard_negative_ctxs: passages that are not related to the question.

To train the DPR and AraELECTRA reader, two public release datasets are used. The format of the dataset matches the format of the well-known SQuAD1.0 dataset (Rajpurkar et al., 2016). For training the DPR, we convert all used datasets from SQuAD structure to DPR structure (see Fig. 3). This structure includes questions, answers, positive passages, and hard negative passages. For every question, 30 hard negative passages are initialized using the BM25 IR passage retrieval. Hard negative examples are the passages that do not contain the answer but match most of the questions’ tokens. Positive passages are the ones that appear in the training set paired with the questions.

### Arabic reading comprehension dataset

ARCD was created by Mozannar et al. (2019) in 2019 and contained 1,395 questions posed by crowd workers on Arabic Wikipedia articles. This dataset was written by professional Arabic speakers.

### TyDiQA

TyDiQA is a multilingual, human-annotated question-answer dataset including typologically diverse languages with 204 thousand question-answer pairs. The data is collected directly from different languages without translation and is written without seeing the answer. The dataset was designed for the training and evaluation of automatic QA systems. The size of the Arabic dataset is 15,645 question-answer pairs. The primary tasks of this dataset are the Passage Selection task (SelectP) and the Minimal Answer Span task (MinSpan). The secondary task is the Gold Passage task (GoldP), which, given a passage that contains the answer, predicts the single contiguous span of letters that answers the question. In this research, we used the Arabic TyDiQA-GoldP dataset (Clark et al., 2020). Table 1 shows the size of the used datasets.

**Table 1:** Arabic reading comprehension datasets.

Reference	Name	Train	Test
(Mozannar et al., 2019)	ARCD	695	700
(Clark et al., 2020)	TyDiQA-GoldP (Arabic)	14,724	921

## End-to-end system: arabic openqa

We built an OpenQA model that employs DPR and the AraELECTRA (Antoun, Baly & Hajj, 2021) passage reader to answer open-domain questions based on Arabic Wikipedia articles. First, the question is passed to the DPR retriever to return the top 20 passages. Then, the candidate passages are fed into the AraELECTRA reader to produce the top three answers. The reader returns an answer span and gives a span score to each passage. The final three answers are chosen from the best span with the highest passage selection score. We use the open-source NLP framework Haystack (Rusic, 2021) for our joint retriever and reader approach.

The probability of a token starting and ending for an answer span and selecting a passage are calculated as follows:

(9) $${P_{{\rm start},i}}(s) = {\rm softmax}{\left( {{{\bf P}_i}{{\bf w}_{{\rm start}}}} \right)_s}$$

(10) $${P_{{\rm end},i}}(t) = {\rm softmax}{\left( {{{\bf P}_i}{{\bf w}_{{\rm end}}}} \right)_t}$$

(11) $${P_{{\rm selected}}}(i) = {\rm softmax}{\left( {{{{ \hat {\bf P}}}^{\top}}{{\bf w}_{{\rm selected}}}} \right)_i}$$

where ${{\bf P}_i} \in {{\rm {\mathbb R}}^{L \times h}}(1 \le i \le k)$ is the reader representation for the *i*-th passage, L is the passage’s maximum length, and h is the hidden dimension.

## Experiments and results

This section details the dataset we used for the experiments on the passage retriever and the reader, including the basic setup.

### Fine tuning multilingual dense passage retriever on Arabic datasets

DPR examines all the documents in the database and then identifies what is relevant and discards what is not. It passes only a small set of candidate documents to the reader. This results in computationally intensive indexing but quick querying.

We fine-tune the multilingual Dense Passage Retriever (mDPR) model from Hugging face (Voidful, 2021) which trained based on multilingual BERT (Devlin et al., 2019). The model is trained on a training set that contains 644,217 multilingual questions and 73,710 questions in the development set. The model trained using the following translated datasets: NQ (Kwiatkowski et al., 2019), Trivia (Joshi et al., 2017), SQuAD (Rajpurkar et al., 2016), Delta Reading Comprehension Dataset (DRCD) (Cui et al., 2020), and MLQA (Lewis et al., 2020). Training the model from scratch requires initializing the embeddings of both the passage and the query models with an Arabic language model and a large Arabic dataset containing enough unique passages. However, applying such a method requires a large amount of well-labeled, clean, non-translated data. Thus, in this step, we decided to fine-tune the mDPR with the ARCD (Mozannar et al., 2019) and TyDiQA-GoldP (Clark et al., 2020) datasets. We used the pre-trained mDPR weights for fine-tuning and initialized both the query and the passage models using the mBERT pre-trained model. We used the previous DPR (Karpukhin et al., 2020) parameters for the maximum passage length, but we reduced the maximum query length to 64 because the queries are rarely longer than 64. We trained the merged and the single training set of the ARCD (Mozannar et al., 2019) and TyDiQA-GoldP (Clark et al., 2020) datasets to 16 epochs since we searched for the best number of train epochs from (4–16), and we used each test set for testing the model.

We implemented our passage retriever module using dense representations, where embeddings are learned from a number of questions and passages using a dual-encoder model. To implement our dense passage retriever, we followed the steps below:

- We used the 01-09-2021 dump of Arabic Wikipedia (Wikimedia Foundation, 2021) as our knowledge source to answer the factoid questions. Only the plain text was extracted and all other data, such as lists and figures, were removed using the WikiExtractor tool (Attardi, 2015). After removing the internal disambiguation, list, index, and outline pages, we were able to extract 3.2 million pages with 2,130,180 articles. Due to memory limitations, we only used 491,253 articles.
- We used Elasticsearch (elastic, 2021) to store the document text and other metadata. We pre-processed by removing empty lines, whitespaces, and long headers and footers. We also split files into small documents of around 100 words, storing these documents in the Elasticsearch document storage. The text’s vector embeddings were indexed based on Elasticsearch Indexing, which was then searched to get answers.
- We initialized our DPR to search for documents in DocumentStore, retrieve some documents, and return the top 20 passages that are most related to the query. Initializing and training the DPR retriever contained the following arguments:

1. document_store: A DocumentStore object from which documents can be retrieved.
2. query_embedding_model: A question encoder checkpoint. We used the mDPR (Voidful, 2021) by hugging-face transformers.
3. passage_embedding_model: A passage encoder checkpoint. We used also the mDPR (Voidful, 2021) by hugging-face transformers.
4. max_seq_len_query: The Maximum number of tokens for the query is 64.
5. max_seq_len_passage: The Maximum number of tokens for the passage is 256. batch_size: The number of queries and passages to encode. The batch size is set to 4.
6. similarity_function: During training, the dot_product function is used to calculate the similarity of the query and passage embeddings.
7. query: The question
8. filters: Contains the dictionary of the keys that indicate a metadata field and the value, which is a list of acceptable values for that field.
9. top_k: Contains the number of passages to retrieve per question.
10. index: Contains the name of the DocumentStore index from which documents can be retrieved.

In our work, we also applied a Term Frequency-Inverse Document Frequency (TF-IDF) document retriever to compare the results to other approaches. In our TF-IDF document retriever, each document is initially preprocessed with the NLTK Arabic tokenizer (Bird et al., 2008) and stop words removal. The TF-IDF weights matrix in the document set, like Arabic Wikipedia, were created using n-gram numbers to take local word order into consideration. The retriever becomes more accurate as the number of documents goes up; however, the retrieving procedure becomes longer and more memory costly. The vector is normalized for every document, and the weights of the TF-IDF vector of question are calculated according to the document’s vocabulary. The score is then calculated as the cosine similarity between the question and vectors of the document. Finally, the top documents with the highest similarity are returned (Mozannar et al., 2019).

**Figure 4:**
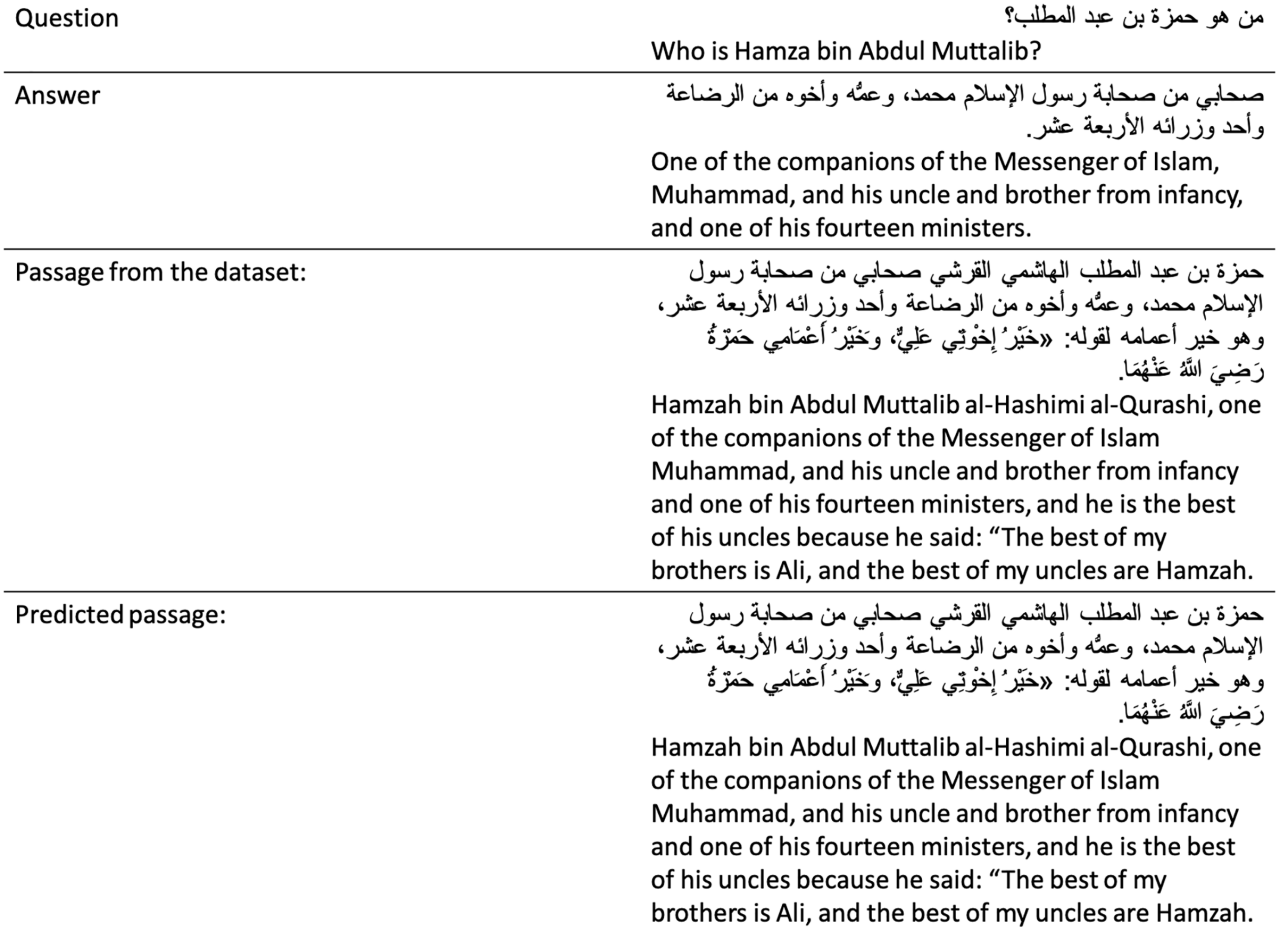
Sample prediction of our DPR from the ARCD test.

**Figure 5:**
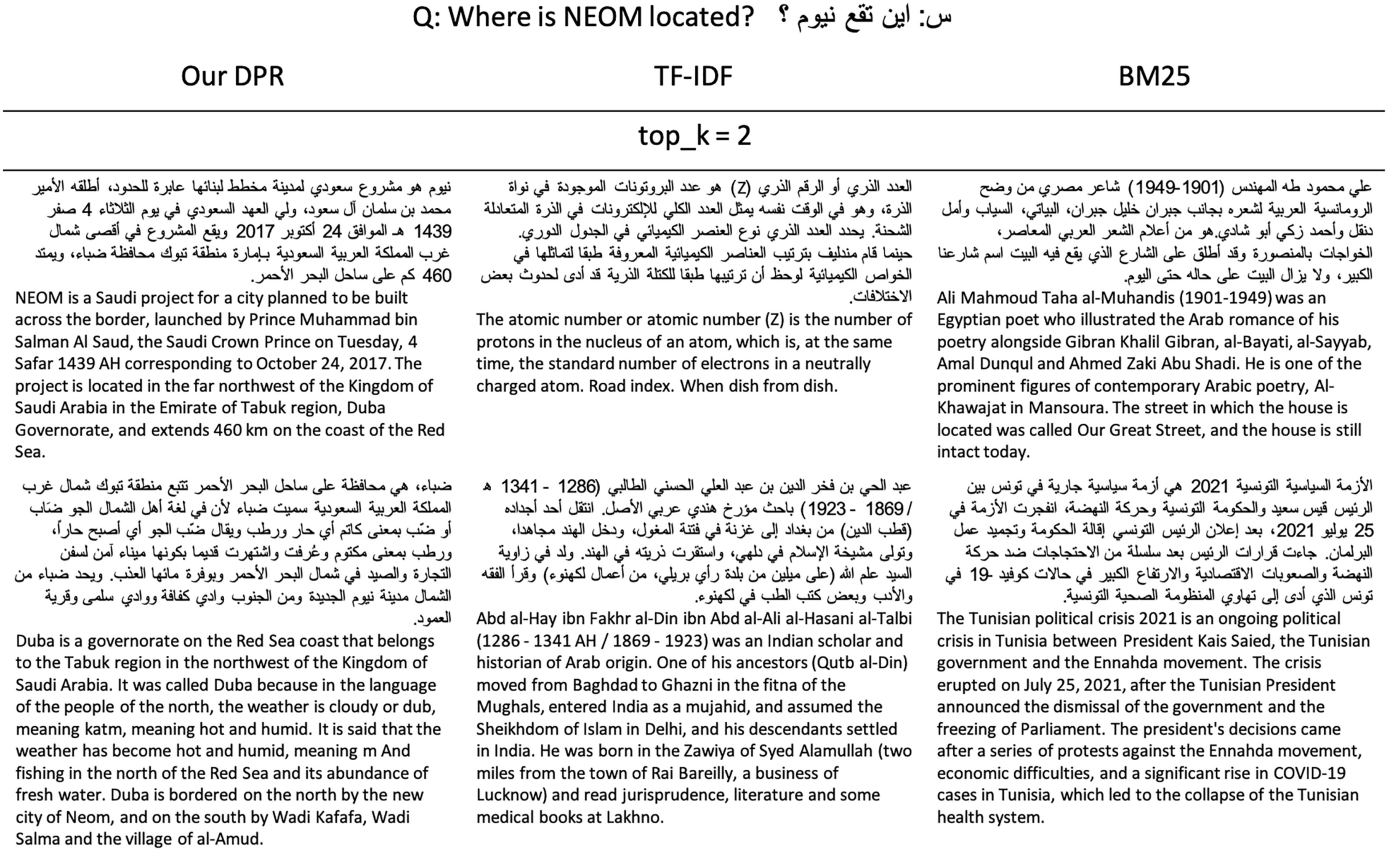
Example of the top two passages retrieved by our DPR, BM25, and TF-IDF.

When evaluated on the TyDiQA-Goldp dev set and the ARCD test set, our dense retriever largely out-performs a TF-IDF in terms of the top-20 and top-100 passage retrieval accuracy and improves our end-to-end OpenQA. Figure 4 shows an example of our DPR prediction. In addition, we run the Elasticsearch’s default BM25 algorithm (elastic, 2021) and the TF-IDF retriever for comparison to get an example of what each retriever retrieves (see Fig. 5).

#### Retriever results

Table 2 shows the results of our DPR trained on combined datasets and a single dataset of ARCD and TyDiQA-GoldP. In all experiments, DPR’s provide higher recall and MAP scores in comparison with traditional methods. Table 3 compares different passage retrieval systems on two Arabic QA datasets, using the top-20 accuracy and top-100 accuracy. Our DPR performs better than the TF-IDF on all datasets. When training with a single dataset, ARCD is limited to a small set of Wikipedia documents, thus causing low results. In contrast, the TyDiQA-GoldP dataset improves the results. Using combined datasets for training improves the accuracy scores in all the experiments.

**Table 2:** Results of the DPR model on TyDiQA-GoldP and ARCD datasets with different training settings.

Training dataset	Testing dataset	Recall	MAP
TyDiQA-GoldP	TydiQA dev set	98.11	93.56
ARCD	ARCD test	96.13	73.68
ARCD+TyDiQA-GoldP	TydiQA dev set	98.00	94.12
ARCD+TyDiQA-GoldP	ARCD test	93.28	68.94

**Table 3:** The results of DPR in comparison with TF-IDF on TydiQA and ARCD datasets.

Model	Training dataset	Test dataset	Dataset size	Accuracy Top-20	Accuracy Top-100
TF-IDF	N/A	TydiQA dev set	921	37.03	48.70
TF-IDF	N/A	ARCD test	696	33.61	40.19
DPR	TyDiQA-GoldP	TydiQA dev set	train:14797 test:917	56.54	62.96
DPR	ARCD	ARCD test	train:684 test:696	46.01	55.41
DPR	ARCD+TyDiQA-GoldP	TydiQA dev set	train:15481 test:917	**58.82**	**65.00**
DPR	ARCD+TyDiQA-GoldP	ARCD test	train:15481 test:696	50.56	57.26

Note: Boldfaced score indicates highest accuracy.

### Fine-tuning AraELECTRA for reading comprehension task

We trained the AraELECTRA on the TyDiQA-GoldP and the ARCD training sets. In the pre-processing step, we applied a pre-processing method that does the following:

- Replace emojis
- Remove HTML markups, except in the TyDiQA-GoldP dataset
- Replace email
- Remove diacritics and tatweel
- Insert whitespaces before and after all non-Arabic digits, English digits, and Arabic and English Alphabet letters
- Insert whitespace between words and numbers or numbers and words

For dataset splitting, we followed the previous work of Antoun, Baly & Hajj (2020) and used the original training and testing set of the ARCD and the TyDiQA-GoldP. We implemented the AraELECTRA-base-discriminator on the reading comprehension datasets, namely the TyDiQA-GoldP set and ARCD. To fine-tune, we searched for the best number of train epochs (2,4,3), and we tried different learning rates [1e−4, 2e−4, 3e−4, 5e−3]. We chose the hyper-parameters that gave us the best results. We used the following hyper-parameters: three epochs and four batch sizes, with a learning rate 3 × 10^−5^. The maximum total input sequence length after WordPiece (Wu et al., 2016) tokenization is 384. The maximum number of tokens for the question is 64, and the maximum length of an answer that can be generated is 30. To provide a valid comparison, we used the same hyperparameters on all experiments.

In the first experiment, we used the ARCD to train the AraELECTRA model. The results, as shown in Table 4, demonstrate a large improvement in our models over the mBERT model. The AraELECTRA achieved the best F1 score and EM. The small size of the ARCD affected the performance of the model. The low results of the ARCD are due to the poor quality of the training examples. The ARCD training set contained text in languages other than Arabic, which can reduce performance due to the unknown words and characters (Antoun, Baly & Hajj, 2021).

**Table 4:** Comparison of two text reader models on ARCD.

Model	ARCD (F1-EM)
mBERT (Mozannar et al., 2019)	50.10–23.9
AraELECTRA (ours)	**68.15–35.47**

Note: Boldfaced score indicates best performance.

In the second experiment of AraELECTRA, we used the TyDiQA-GoldP dataset. In this experiment, we obtained a better result for the F1 score and EM compared to the ARCD that used the same model (see Table 5). We recorded an increase in the exact match score over the ARCD. EM measures the percentage of predictions that match any of the ground truth.

**Table 5:** Comparison of the different text reader models on TyDiQA-GoldP.

Model	TyDiQA-GoldP (F1-EM)
mBERT (Clark et al., 2020)	81.7–
AraBERTv0.1 (Antoun, Baly & Hajj, 2020)	82.86–68.51
AraBERTv1 (Antoun, Baly & Hajj, 2020)	79.36–61.11
AraBERTv0.2-base (Antoun, Baly & Hajj, 2020)	85.41–73.07
AraBERTv2-base (Antoun, Baly & Hajj, 2020)	81.66–61.67
AraBERTv0.2-large (Antoun, Baly & Hajj, 2020)	86.03–73.72
AraBERTv2-large (Antoun, Baly & Hajj, 2020)	82.51–64.49
ArabicBERT-base (Antoun, Baly & Hajj, 2020)	81.24–67.42
ArabicBERT-large (Antoun, Baly & Hajj, 2020)	84.12–70.03
Arabic-ALBERT-base (Antoun, Baly & Hajj, 2020)	80.98–67.10
Arabic-ALBERT-large (Antoun, Baly & Hajj, 2020)	81.59–68.07
Arabic-ALBERT-xlarge (Antoun, Baly & Hajj, 2020)	84.59–71.12
AraELECTRA (Antoun, Baly & Hajj, 2020)	**86.86–74.91**
AraELECTRA (ours)	86.01–74.07

Note: Boldfaced score indicates best performance.

**Figure 6:**
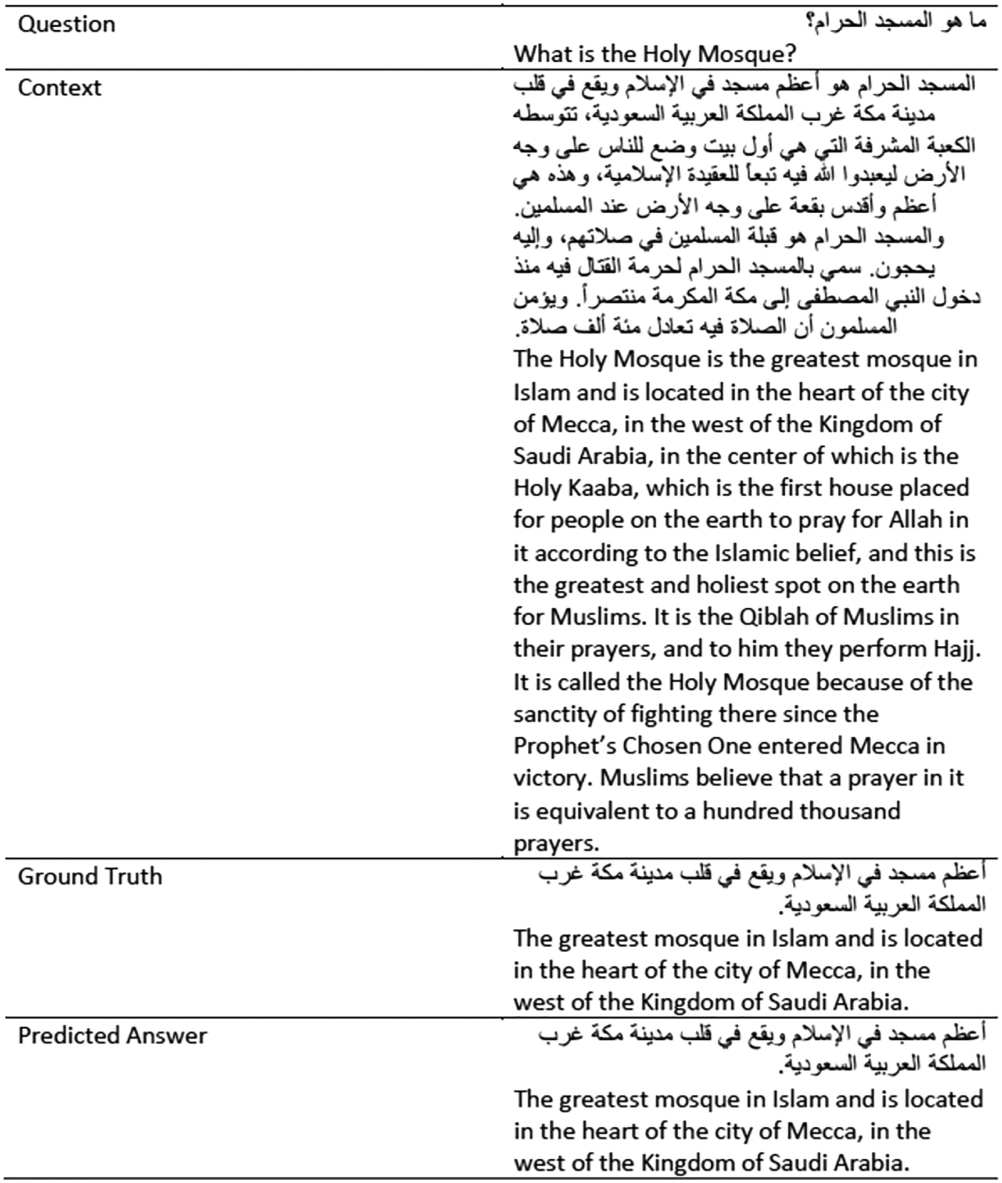
Example of results from the TyDiQA-GoldP development set.

In our OpenQA system, we used the AraELECTRA based on the TyDiQA-GoldP dataset because the results were much higher than those of the ARCD dataset. We believe that this is because the dataset is much cleaner and is correctly labeled, without any translations (Clark et al., 2020). This dataset was created by experts in the Arabic language. We recognize that a deep understanding of the data itself is key to understanding what modeling techniques are best suited for the data. Running those experiments was computationally high, and the model took more than 12 h to train only three epochs. In Fig. 6, we capture one of the results from the TyDiQA-GoldP development set. It can be seen that the predicted answer exactly matches the exact ground truth answer.

### Final results

Our system focuses on answering questions using Arabic Wikipedia. We used 491,253 documents to build an OpenQA system that can answer any type of factoid question where the answer can be found on and retrieved from Wikipedia. Table 6 compares our OpenQA and SOQAL (Mozannar et al., 2019) system on the ARCD dataset, using the top-1, top-3, and top-5 answers. Our OpenQA system achieves better results than the SOQAL system. We conclude that using deep learning techniques in all modules will improve the results.

**Table 6:** Comparison of our OpenQA and SOQAL when returning the top k answers.

Model	Evaluation dataset	EM	F1
SOQAL (top-1) (Mozannar et al., 2019)	ARCD	12.8	27.6
SOQAL (top-3) (Mozannar et al., 2019)	ARCD	17.8	37.9
SOQAL (top-5) (Mozannar et al., 2019)	ARCD	20.7	42.5
our OpenQA (top-1)	ARCD	15.7	36.4
our OpenQA (top-3)	ARCD	23.1	39.6
our OpenQA (top-5)	ARCD	**26.8**	**43.1**

Note: Boldfaced score indicates best performance.

Table 7 summarizes our final end-to-end QA results, measured by the F1 score and EM, with the aspects of different training datasets of DPR for the passage retriever. Table 7 shows how increased retriever accuracy usually leads to better QA results in every dataset. In addition, the models perform well when evaluated to the TyDiQA-GoldP dataset. However, the ARCD dataset performs poorly in multi and single-training settings.

**Table 7:** End-to-end QA results. Our DPR is trained using single or merged training datasets, as indicated by the terms single and multi.

Training setting	Model	Evaluation dataset	EM	F1
Single	ORQA (Lee, Chang & Toutanova, 2019)	NQ	33.3	–
		TriviaQA	45.0	
		WQ	36.4	
		TREC	30.1	
		SQuAD	20.2	
Single	REALM (Guu et al., 2020)	NQ	39.2	–
		WQ	40.2	
		TREC	46.8	
Single	DPR (Karpukhin et al., 2020)	NQ	41.5	–
		TriviaQA	56.8	
		WQ	34.6	
		TREC	25.9	
		SQuAD	29.8	
Single	DPR + BM25 (Karpukhin et al., 2020)	NQ	39.0	–
		TriviaQA	57.0	
		WQ	35.2	
		TREC	28.0	
		SQuAD	36.7	
Multi	DPR (Karpukhin et al., 2020)	NQ	41.5	–
		TriviaQA	56.8	
		WQ	42.4	
		TREC	49.4	
		SQuAD	24.1	
Multi	DPR + BM25 (Karpukhin et al., 2020)	NQ	38.8	–
		TriviaQA	57.9	
		WQ	41.1	
		TREC	50.6	
		SQuAD	35.8	
Single	our DPR	TyDiQA-GoldP	41.8	50.1
Single	our DPR	ARCD	15.1	35.3
Multi	our DPR	TyDiQA-GoldP	43.1	51.6
Multi	our DPR	ARCD	15.7	36.3

## Conclusions

OpenQA is an important research area in the NLP field. The goal of a QA system is to answer any questions written in a natural language. The current growth of language models like BERT and ELECTRA has made it possible for all kinds of NLP tasks to make significant progress. In this paper, we evaluate the performance of an OpenQA system using the DPR and the AraELECTRA models in the Arabic language. Our paper addresses the problem of Arabic OpenQA and how different factors like datasets will affect the results. For initializing our OpenQA system, a model is trained to answer questions from the retrieved passages. The DPR and the AraELECTRA passage reader were trained in the context of QA with ARCD and TyDiQA-GoldP datasets. Our DPR outperforms the traditional TF-IDF information retriever in terms of top-20 and top-100 passage retrieval accuracy and improves our end-to-end QA system. For future work, the retriever can be improved by combining DPR with BM25 or other IR models using a hybrid approach.

## Supplemental Information

10.7717/peerj-cs.952/supp-1 Supplemental Information 1 ARCD dataset.

10.7717/peerj-cs.952/supp-2 Supplemental Information 2 ARCD+TYDIQA Dataset.

10.7717/peerj-cs.952/supp-3 Supplemental Information 3 Code for combining retriever with reader.

## Additional Information and Declarations

### Data Availability

The following information was supplied regarding data availability: The TyDiQA dataset is available at GitHub: https://github.com/google-research-datasets/tydiqa. The ARCD dataset is available at https://huggingface.co/datasets/arcd.

## References

[ref-1] Ahmed W, Ahmed A, Babu Anto P (2017). Web-based Arabic question answering system using machine learning approach. International Journal of Advanced Research in Computer Science.

[ref-2] Ahmed W, Babu Anto P (2016). Answer extraction for how and why questions in question answering systems. International Journal of Computational Engineering Research (IJCER).

[ref-3] Ahmed W, Bibin PA, Babu Anto P (2017). Question answering system based on neural networks. International Journal of Engineering Research.

[ref-4] Almiman A, Osman N, Torki M (2020). Deep neural network approach for Arabic community question answering. Alexandria Engineering Journal.

[ref-5] Amati G, Liu L, Özsu MT (2009). BM25. Encyclopedia of Database Systems.

[ref-6] Antoun W, Baly F, Hajj H (2021). AraELECTRA: pre-training text discriminators for Arabic language understanding.

[ref-7] Antoun W, Baly F, Hajj H (2020). AraBERT: transformer-based model for Arabic language understanding.

[ref-8] Attardi W (2015). Wikiextractor. GitHub. https://github.com/attardi/wikiextractor.

[ref-9] Bird S, Klein E, Loper E, Baldridge J (2008). Multidisciplinary instruction with the natural language toolkit.

[ref-37] Briggs J (2021). How Dense Passage Retrievers (DPR) Work. https://towardsdatascience.com/how-to-create-an-answer-from-a-question-with-dpr-d76e29cc5d60.

[ref-10] Chen D, Fisch A, Weston J, Bordes A (2017). Reading Wikipedia to answer open-domain questions.

[ref-11] Clark JH, Choi E, Collins M, Garrette D, Kwiatkowski T, Nikolaev V, Palomaki J (2020). TyDi QA: a benchmark for information-seeking question answering in typologically diverse languages. Transactions of the Association for Computational Linguistics.

[ref-12] Clark K, Luong MT, Le QV, Manning CD (2020). Electra: Pre-training text encoders as discriminators rather than generators. arXiv preprint.

[ref-13] Cui Y, Liu T, Yang Z, Chen Z, Ma W, Che W, Wang S, Hu G (2020). A sentence cloze dataset for Chinese machine reading comprehension.

[ref-14] Devlin J, Chang MW, Lee K, Toutanova K (2019). BERT: pre-training of deep bidirectional transformers for language understanding.

[ref-15] elastic (2021). Free and open search: the creators of elastic search, elk & kibana | elastic. https://www.elastic.co/.

[ref-16] Guu K, Lee K, Tung Z, Pasupat P, Chang MW (2020). Realm: retrieval-augmented language model pre-training. arXiv preprint.

[ref-17] Hedderich MA, Lange L, Adel H, Strötgen J, Klakow D (2021). A survey on recent approaches for natural language processing in low-resource scenarios.

[ref-18] Huang Z, Xu S, Hu M, Wang X, Qiu J, Fu Y, Zhao Y, Peng Y, Wang C (2020). Recent trends in deep learning based open-domain textual question answering systems. IEEE Access.

[ref-19] Johnson J, Douze M, Jegou H (2021). Billion-scale similarity search with GPUs. IEEE Transactions on Big Data.

[ref-20] Joshi M, Choi E, Weld D, Zettlemoyer L (2017). TriviaQA: a large scale distantly supervised challenge dataset for reading comprehension.

[ref-21] Karpukhin V, Oguz B, Min S, Lewis P, Wu L, Edunov S, Chen D, Yih WT (2020). Dense passage retrieval for open-domain question answering.

[ref-22] Kwiatkowski T, Palomaki J, Redfield O, Collins M, Parikh A, Alberti C, Epstein D, Polosukhin I, Devlin J, Lee K, Toutanova K, Jones L, Kelcey M, Chang M-W, Dai AM, Uszkoreit J, Le Q, Petrov S (2019). Natural questions: a benchmark for question answering research. Transactions of the Association for Computational Linguistics.

[ref-23] Lee K, Chang MW, Toutanova K (2019). Latent retrieval for weakly supervised open domain question answering.

[ref-24] Lewis P, Oguz B, Rinott R, Riedel S, Schwenk H (2020). MLQA: evaluating cross-lingual extractive question answering.

[ref-25] Mozannar H, Maamary E, El Hajal K, Hajj H (2019). Neural Arabic question answering.

[ref-26] Qiu XP, Sun TX, Xu YG, Shao YF, Dai N, Huang XJ (2020). Pre-trained models for natural language processing: a survey. Science China Technological Sciences.

[ref-27] Rajpurkar P, Zhang J, Lopyrev K, Liang P (2016). SQuad: 100,000+ questions for machine comprehension of text.

[ref-28] Rusic M (2021). Nlp solutions to streamline neural search and question answering | deepset. https://www.deepset.ai/.

[ref-29] Sammut C, Webb GI (2017). Encyclopedia of machine learning and data mining.

[ref-30] Teufel S (2007). An overview of evaluation methods in trec ad hoc information retrieval and trec question answering. Evaluation of Text and Speech Systems.

[ref-31] Voidful (2021). voidful/dpr ctx encoder bert base multilingual model nlp hub. https://huggingface.co/voidful/.

[ref-32] Wikimedia Foundation (2021). arwiki dump progress on 20210901. https://archive.org/details/arwiki-20211220.

[ref-33] Wu Y, Schuster M, Chen Z, Le QV, Norouzi M, Macherey W, Krikun M, Cao Y, Gao Q, Macherey K, Klingner J, Shah A, Johnson M, Liu X, Kaiser Ł, Gouws S, Kato Y, Kudo T, Kazawa H, Stevens K, Kurian G, Patil N, Wang W, Young C, Smith J, Riesa J, Rudnick A, Vinyals O, Corrado G, Hughes M, Dean J (2016). Google’s neural machine translation system: bridging the gap between human and machine translation. ArXiv preprint.

[ref-34] Yang W, Xie Y, Lin A, Li X, Tan L, Xiong K, Li M, Lin J (2019). End-to-end open-domain question answering with BERTserini.

[ref-35] Zhang Z, Liu S, Li M, Zhou M, Chen E (2018). Bidirectional generative adversarial networks for neural machine translation.

[ref-36] Zhu F, Lei W, Wang C, Zheng J, Poria S, Chua TS (2021). Retrieving and reading: a comprehensive survey on open-domain question answering. ArXiv preprint.

